# Elaborating the potential of Artificial Intelligence in automated CAR-T cell manufacturing

**DOI:** 10.3389/fmmed.2023.1250508

**Published:** 2023-09-21

**Authors:** Niklas Bäckel, Simon Hort, Tamás Kis, David F. Nettleton, Joseph R. Egan, John J. L. Jacobs, Dennis Grunert, Robert H. Schmitt

**Affiliations:** ^1^ Fraunhofer Institute for Production Technology IPT, Aachen, Germany; ^2^ Institute for Computer Science and Control, Hungarian Research Network, Budapest, Hungary; ^3^ IRIS Technology Solutions, Barcelona, Spain; ^4^ Department of Biochemical Engineering, Mathematical Modelling of Cell and Gene Therapies, University College London, London, United Kingdom; ^5^ Clinical Care and Research, ORTEC B.V., Zoetermeer, Netherlands; ^6^ Laboratory for Machine Tools and Production Engineering (WZL), RWTH Aachen University, Aachen, Germany

**Keywords:** CAR-T manufacturing, artificial intelligence, machine learning, cell and gene therapy, immunotherapy, data analytics, ATMP, advanced therapy

## Abstract

This paper discusses the challenges of producing CAR-T cells for cancer treatment and the potential for Artificial Intelligence (AI) for its improvement. CAR-T cell therapy was approved in 2018 as the first Advanced Therapy Medicinal Product (ATMP) for treating acute leukemia and lymphoma. ATMPs are cell- and gene-based therapies that show great promise for treating various cancers and hereditary diseases. While some new ATMPs have been approved, ongoing clinical trials are expected to lead to the approval of many more. However, the production of CAR-T cells presents a significant challenge due to the high costs associated with the manufacturing process, making the therapy very expensive (approx. $400,000). Furthermore, autologous CAR-T therapy is limited to a make-to-order approach, which makes scaling economical production difficult. First attempts are being made to automate this multi-step manufacturing process, which will not only directly reduce the high manufacturing costs but will also enable comprehensive data collection. AI technologies have the ability to analyze this data and convert it into knowledge and insights. In order to exploit these opportunities, this paper analyses the data potential in the automated CAR-T production process and creates a mapping to the capabilities of AI applications. The paper explores the possible use of AI in analyzing the data generated during the automated process and its capabilities to further improve the efficiency and cost-effectiveness of CAR-T cell production.

## 1 Introduction

The approval of the first chimeric antigen receptor (CAR)-T cell product in the European Union in 2018 marked a significant paradigm shift in the treatment of acute lymphoblastic leukemia (ALL) (EMA/188757/2022 Kymriah, 2022). Since then, the field of advanced therapies has rapidly evolved, with the approval of nine additional Gene Therapy Medicinal Products (GTMP) and a multitude of ongoing clinical trials. Approved GTMPs are for the treatment of multiple myeloma, melanoma and inherited diseases such as hemophilia and retinal dystrophy (Paul-Ehrlich-Institut, 2023). In addition, current clinical trials focus on solid tumors and alternatives for T cells such as NK cells and macrophages (Marofi et al., 2021; Pan et al., 2022). However, despite the significant clinical success of these therapies, high costs in manufacturing and supply hinder wide-scale patient access. For cost reduction, the complex manufacturing processes need to be better characterized to ultimately ensure a successful therapy outcome.

For this reason, the field is moving steadily toward digitization and automation of the entire therapy process (Blache et al., 2022). One project dedicated to this approach is the European Union Horizon 2020 project AIDPATH (European Commision, 2021a) which is an acronym for Artificial Intelligence-driven, Decentralized Production for Advanced Therapies in the Hospital. AIDPATH aims to develop an open platform for the production of CAR-T cells using flexible automation concepts together with digital solutions for data management and the integration of AI (Hort et al., 2022). In particular, the use of AI holds great potential and has the possibility to improve CAR-T cell manufacturing in the future. AI has gained increasing popularity in recent years due to its ability to process ever-increasing amounts of data and support its analytical capabilities.

The use of AI in CAR-T cell therapy presents both opportunities and challenges. Integrating AI technologies can improve manufacturing efficiency and accuracy, optimize logistics, and reduce costs. AI can also assist in identifying appropriate patients for therapy and help monitor therapy progression and predict treatment responses. However, there are still open issues and challenges to overcome. Privacy, security, and ethical issues play a critical role in implementing AI in CAR-T cell therapy. In addition, the integration of AI systems into existing production workflows and the validation of AI-based decisions still need to be explored.

Therefore, this paper is dedicated to the topic of AI in CAR-T cell therapy. It highlights the fundamentals and potentials of AI in a manufacturing context and explores why its use in CAR-T cell therapy has been limited to date. Furthermore, this paper discusses the potential uses of AI in the treatment process and identifies existing barriers. In addition, existing AI methods are categorized and listed along the therapy process. Finally, an outlook on the future development of AI in the field of CAR-T cell therapy is provided, highlighting potential trends and opportunities.

Overall, the integration of AI into CAR-T cell therapy has the potential to provide significant advances in the production of CAR-T cells and treatment of leukemia and lymphoma. By overcoming challenges and targeting the potential of AI, new therapies can be developed more efficiently and made available to patients more quickly.

## 2 Definition of AI and applications in manufacturing

The potential of AI in healthcare is enormous, as evidenced by its rapid market growth and significant investments in research and development. By 2027, the AI market is projected to reach a staggering $407 billion, with the manufacturing sector poised to experience a financial impact of $3.8 trillion by 2035 (Maslej et al., 2023). Notably, the healthcare industry has received the highest investment, amounting to $6.1 billion in 2022. Organizations that have already embraced AI in healthcare have reported remarkable cost reductions and revenue increases (Haan, 2023).

In information systems, AI can be described as an agent. Kühl et al. distinguish here between simple reflex agents and learning agents (Kühl et al., 2022). A reflexive agent applies knowledge once acquired from an initial implementation to its environment, while a learning agent continues to learn by interacting with its environment after initial training. Both types of agents are described by their interaction with their environment. This interaction consists of the reception of data from the environment and on an action to be executed in the environment. Internally, acquired knowledge is applied to achieve a given goal by the execution of an action. Now, such an intelligent agent may have acquired this knowledge by training Machine Learning (ML) models, or it may have a non-ML based knowledge representation, such as a rule-based expert system. ML, meanwhile, can be viewed as an implementation of statistical learning. Thus, ML, is a method applied by AI systems (Kühl et al., 2020).

Such an intelligent agent can interact with its environment with different degrees of autonomy. A possible categorization of autonomy can be made by the amount of human interaction in the process of data analysis from the data basis to the decision or action. Here, a distinction can be made between descriptive, diagnostic, predictive, and prescriptive tasks with which the agent is entrusted (Sallam et al., 2014; Kühn et al., 2018). A descriptive agent describes what is happening in the environment. The human must figure out why it is happening and what will happen to derive a decision or action that will change the environment in the desired sense. A diagnostic agent now goes one step further and tries to explain relationships in the environment. A predictive agent goes further still and predicts how the environment will change in the future. Finally, a prescriptive agent supports the human in deciding which action to take to achieve a desired result or carries out the action itself. A bioreactor can provide an example of the differentiation of agents in the process of CAR-T cell production explained here: a descriptive agent describes the number of cells in the bioreactor, a diagnostic agent can justify why exactly this number of cells is found in the reactor on the basis of the information supplied. A predictive agent can predict the number of cells for a point in time in the future and a prescriptive agent can determine the optimal time to harvest and propose it to the operator and if all regulatory aspects are covered, trigger the process itself.

## 3 CAR-T therapy process and its challenges

**FIGURE 1:**
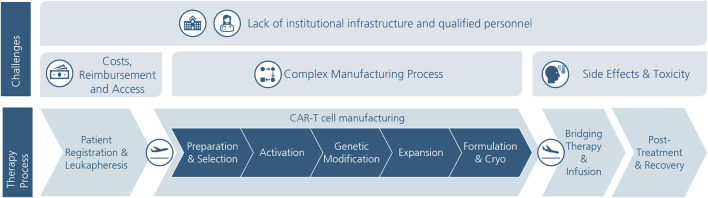
CAR-T cell therapy process and its challenges.

The manufacturing and provision of CAR-T cells pose new challenges for hospitals and treatment centers. Due to the autologous nature of the therapy, T cells are removed from patients in the hospital, shipped to a pharmaceutical company or an academic site for CAR-T cell manufacturing, and then shipped back for administration to the patient. Figure 1 illustrates the treatment process and the challenges involved (Iyer et al., 2018; Enejo, 2019; Braga et al., 2021).

First, the patients are registered in the hospital and their eligibility for the therapy is determined (Braga et al., 2021). Blood is then drawn from the patient and the leukocytes are isolated (leukapheresis). At the manufacturing site the leukocytes are preprepared and the desired T cells are selected. Which T cells are selected depends on the chosen product. Which T cells and in which ratio they yield the best quality is the focus of current research. In the subsequent activation step, the cells are stimulated for proliferation and differentiation. Afterward, the CAR is integrated in the genome of the T cells (genetic modification). Different methods can be used for this such as viral transduction or non-viral transfection. The latter was developed more recently for safer and more cost-efficient genetic modification (Harris and Elmer, 2021). Then, the CAR-T cells are expanded to reach the required amount. With 7–10 days, the expansion process is by far the longest manufacturing process and thus a major driver for the overall delivery time, besides the final quality and release criteria control. Therefore the trend is to reduce the duration of the expansion time to the minimum amount of time to get a sufficient product and reduce the delivery time. Lastly, the CAR-T cells are cryopreserved and shipped back to the hospital. At the hospital, the patient receives the necessary bridging therapies (e.g., chemotherapy), the manufactured product is checked and administered to the patient. In the post-treatment phase, the patient continues to be monitored and remains in the hospital for up to 10 days. For the following 28 days, it is recommended that the patient stays within a 2-h distance to the hospital (Kymriah, 2018; Iyer et al., 2018; Vormittag et al., 2018; Braga et al., 2021).

Across the treatment process, challenges emerge that currently still hinder equitable and affordable CAR-T cell therapy. Figure 1 summarizes the main challenges. A major barrier to wide access to CAR-T cell therapy is the associated cost. The cost of approved products is $475,000 for Kymriah® and $373,000 for Yescarta® (Geethakumari et al., 2021). In addition, there are other costs associated with bridging therapies, follow-up, and possible treatment of side effects (Kamal-Bahl et al., 2022). In the EU, reimbursement practices for CAR-T cell therapies are inconsistent and occur through separate compensation payments. Pricing decisions are mostly made between pharmaceutical companies and regulators. A uniform reimbursement model is proving difficult due to regional and country-specific factors (Haag et al., 2022). A 2020 study highlights the significant administrative and financial challenges faced by hospitals and treatment centers in Germany. Problems with reimbursement and the need to make advance payments are often apparent here (Wörmann, 2020). One solution for uniform and fair reimbursement could be outcome-based reimbursement models (OMS), in which costs are only incurred if the therapy is successful. Challenges arise here, however, in the comparability of clinical studies and an overall lack of understanding of the manufacturing process (Solbach et al., 2020).

An autologous CAR-T cell product is a complex biological product consisting of the patient’s genetically modified T cells. Accordingly, the quality of the product varies greatly with the patient’s biological material as well as with the manufacturing process. Thus, even small effects in the process can have a large impact on the product. These include, for example, different procedures for T-cell stimulation and the gene delivery process (Stock et al., 2019), as well as the choice of reagents (Egri et al., 2020; Ghassemi et al., 2020). The focus in recent years has also tended to be on optimizing biological parameters to increase response rates rather than improving the overall process chain. More recently, the field has also been shifting to optimizing the production process and thus reducing process times and eliminating manual processes. Technological concepts and devices enable the automation of single process steps (e.g., through liquid handling units or bioreactors) and the entire process chain (e.g., CliniMACS^®^, Lonza Cocoon^®^) (Moutsatsou et al., 2019). While the latter drastically reduce human interaction and thus increase standardization and reproducibility, they follow a one-device-per-patient approach, which makes scalability difficult. In the AIDPATH research project, these limitations are being addressed via a modular, vendor-independent platform for parallel, automated manufacturing and quality control (Hort et al., 2022).

Another challenge is evident in the side effects and uncertain efficacy of CAR-T cell therapy. The most common side effects are cytokine release syndrome (CRS) and immune effector cell-associated neurotoxicity syndrome (ICANS). In CRS, there is a massive release of cytokines caused by the contact of CAR-T cells with the target antigens of cancer cells. ICANS affects the central nervous system and can cause a variety of symptoms. Other phenomena that affect efficacy include antigen loss, tumor heterogeneity, and lack of persistence (Ayuketang et al., 2022; Rees et al., 2022).

Adequate infrastructure also has a major impact on equitable access to CAR-T cell therapy. While there is sufficient coverage in Germany with 39 CAR-T centers (Novartis, 2023), there are large gaps in coverage in the USA (especially in the Southeast and Midwest) (Kamal-Bahl et al., 2022). This involves not only the buildings, facilities, and cleanrooms, but also adequately trained personnel. A variety of individuals from different disciplines are needed throughout the therapy process, all of whom must be trained and qualified (Beaupierre et al., 2019).

## 4 AI application scenarios in CAR-T cell therapy

In this section the process described in Section 3 is overlaid with AI use cases found in the literature. Table 1 provides an overview of the process steps as well as the stages of development of AI systems. Relevant work is mapped herein to identify focus areas of research and highlight potential gaps.

**TABLE 1:** Relevant AI research in CAR-T cell manufacturing and therapy (* marks work, that is not yet implemented).

	CAR design	Patient evaluation and selection	T-Cell extraction and preparation	Genetic engineering and expansion	Conditioning therapy and infusion	Post-treatment and recovery
descriptive	Lee et al. (2020)		Naghizadeh et al. (2022)	[UC2]		
diagnostic		Liberini et al. (2021), Beekers et al. (2023)				
predictive	Mösch et al. (2019), Dannenfelser et al. (2020), Lee et al. (2020)	Gil and Grajek (2022) ^*^	O'Reilly et al. (2023)	[UC2] Wu et al. (2018), Reyes et al. (2022)		Banerjee et al. (2021), Tang et al. (2020), Tedesco and Mohan (2021), Le et al. (2019), Fleuren et al. (2020), Bedoya et al. (2020), Giannini et al. (2019), Beekers et al. (2023)
prescriptive			[UC3, 4] Sugimoto (2019)	[UC1, 3, 4]	[UC4]	

A large focus of current research on AI in CAR-T cell therapy deals with patient follow-up. Here, the emphasis is on predicting the occurrence of side effects like CRS or sepsis after the therapy is administered (Bedoya et al., 2020; Fleuren et al., 2020; G et al., 2019; Le et al., 2019; Tang et al., 2020; Tedesco and Mohan, 2021). To monitor patients more closely, one team is proposing the use of smart devices and wearables to use ML to analyse the data collected there and respond even more quickly (Banerjee et al., 2021). In the field of patient evaluation and selection, biomarker evaluation plays a crucial role to ensure successful therapy in the CAR-T process. In this regard (Gil and Grajek, 2022), suggests a consideration of biomarker-based selection criteria to ensure that therapy is optimally effective (not yet implemented, therefore marked with * in Table 1). Another use case is to select patients in whom the therapy is likely to achieve the best results (Liberini et al., 2021). Another important step in the CAR-T process is the extraction and preparation of the T cells. Here, healthy CD3 T cells are specifically selected to provide an optimal starting point for the further steps of the process (Sugimoto, 2019). In addition, pre-cell selection data will allow prediction of optimal cell selection timing for patients individually to achieve maximum benefit (O'Reilly et al., 2023).

In the genetic engineering and expansion phase, predictive quality assessment of the cell product is performed to predict the clinical outcome of the therapy (Naghizadeh et al., 2022). Surveys by Wu et al. (Wu et al., 2018) in 2018 and Reyes et al. (Reyes et al., 2022) in 2022 provide insights into the state-of-the-art soft sensors and AI for cell culture control. Wu et al. (Wu et al., 2018) focus on automated cell expansion trends and KPIs such as foaming, cell count, viability, glycosylation, biomass, and morphology, highlighting fluorescence, Raman spectroscopy, chemometrics, and artificial neural networks. Reyes et al. (Reyes et al., 2022) conduct a comprehensive survey covering various modern sensor tools, including artificial neural networks, spectroscopy, optical sensors, free-floating wireless sensors, and statistical methods for modeling cell density and antibody titers. Another field that is being strongly addressed is the design of the CAR gene and its effect on cells and tumours prior to the manufacturing process. Here, the correlations between different possible markers and their effects on tumour cells are investigated and an attempt is made to predict possible efficacy (Mösch et al., 2019; Dannenfelser et al., 2020; Lee et al., 2020).

In addition to the listed use cases from literature, other use cases for AI in CAR-T cell production are being investigated in the AIDPATH research project. Two of those use cases (UC) deal directly with the most time-consuming process step, the expansion of the CAR-T cells in the bioreactor. Use case 1 focuses on the development of a digital twin of the bioreactor by mechanistically modelling its design and control, as well as modelling the CAR-T cells growth via the consumption of key nutrients and production of metabolites. This digital twin will provide a soft-sensor of cell-concentration in real-time, as well as short term (1–2 days) forecasts of cell concentration in the future. Such predictions can then be used to inform when the expansion stage should be terminated based on assessment of whether the target dose (i.e., required cell number for treatment) has been reached. In Use case 2 a reactive online process control based on a set of ‘soft’ sensors is developed to complement the existing PID controller for real-time monitoring of key bioreactor parameters [UC2]. These soft sensors process data from 8 selected ‘hard’ sensors and provide consensus alerts to the human operator. Different soft sensor algorithms, including statistically based and artificial intelligence techniques, contribute to the overall confidence in assessing the situation. Future developments aim to include patient-specific adaptations by adjusting sensor set points and algorithm configurations. Furthermore, the modular concept (Section 3) raises the problem of the production scheduling of the manufacturing platform. If, in the future, the capacity of the plant is increased so that the products of multiple patients can be manufactured concurrently, the optimization of the production through scheduling [UC3] becomes inevitable. The uncertainty of the cell-expansion process combined with hard time constraints between consecutive production processes requires new scheduling methodology. Furthermore, the coordination of the patients’ therapies running in parallel [UC4] must be added to the system in order to manage the uncertainties in all steps of the therapies and to ensure that the patient and the product are ready at the same time (Hort et al., 2022).

A further consideration in the project is the personalizable nature of the CAR-T cell product. Different patients theoretically require personalized product properties, such as CD4/CD8 ratio or similar. These are balanced on competing risks, e.g., tumour-free survival and therapy survival. In addition, the accompanying therapy must be adapted to the patient. Here, a clinical decision support system can provide support [UC5] (Beekers et al., 2023).

## 5 Conclusion and outlook

In this paper, a classification of AI systems for the application in CAR-T cell manufacturing and therapy was proposed and filled with approaches from literature and current investigations in AIDPATH. Even if this paper is only intended to provide an initial overview and makes no claim to completeness, it is nevertheless possible to draw initial conclusions and derive suggestions for the further use of AI in CAR-T therapy. While the first ML algorithms exist in the processes upstream and downstream of the manufacturing process—CAR design and post-treatment—there is still a lack of approaches to control and optimize the manufacturing process as such. The authors see the reason for this in the lack of understanding between the effects of the critical process parameters (CPP) and the critical quality attributes (CQA). And it is precisely at this point that AI systems can release their full potential through comprehensive data analyses and determine cause-and-effect relationships (diagnostic). In addition to the technical implementation of such AI systems, the authors see in particular the need for (EMA/188757/2022 Kymriah, 2022) knowledge transfer between data scientists, biotechnologists, and physicians (Paul-Ehrlich-Institut, 2023), adapting regulatory processes based on adaptive manufacturing and Quality by Design approaches and (Marofi et al., 2021), an end-to-end, standardized data acquisition and provision. International EU consortia such as AIDPATH (European Commision, 2021a), ImSavar (European Commision, 2019) and T2EVOLVE (European Commision, 2021b), have set themselves the task of addressing these needs and aim at an equitable and affordable access to CAR-T cell therapy.

## Data Availability

The original contributions presented in the study are included in the article/Supplementary material, further inquiries can be directed to the corresponding author.

## References

[B1] Ayuketang F. A. Ulrich J. (2022). “Management of cytokine release syndrome (CRS) and HLH,” in The EBMT/EHA CAR-T cell handbook. Editors Kröger N. Gribben J. Chabannon C. Yakoub-Agha I. Einsele H. (Cham: CH), 135–140. 36122067

[B2] Banerjee Rahul Shah Nina Dicker Adam P. (2021). Next-generation implementation of chimeric antigen receptor T-cell therapy using digital health. JCO Clin. Cancer Inf. 5 (5), 668–678. 10.1200/CCI.21.00023 34110929

[B3] Beaupierre A. Kahle N. Lundberg R. Patterson A. (2019). Educating multidisciplinary care teams, patients, and caregivers on CAR T-cell therapy. J. Adv. Pract. Oncol. 10 (3), 29–40. 10.6004/jadpro.2019.10.4.12 33520344 PMC7521122

[B4] Bedoya A. D. Futoma J. Clement M. E. Corey K. Brajer N. Lin A. (2020). Machine learning for early detection of sepsis: an internal and temporal validation study. Jamia Open 3 (2), 252–260. 10.1093/jamiaopen/ooaa006 32734166 PMC7382639

[B5] Beekers I. verkouter i. Vegelien A. Velazquez S. Juan M. Sanges C. (2023). Mathematical optimization of personalized CAR-T cell products: Mathematical approach towards personalized prediction of the mostefficient CAR-T cell product using survival analysis with competing risks. Germany: 5th European CAR T-cell meeting.

[B6] Blache U. Popp G. Dünkel A. Koehl U. Fricke S. (2022). Potential solutions for manufacture of CAR T cells in cancer immunotherapy. Nat. Commun. 13 (1), 5225. 10.1038/s41467-022-32866-0 36064867 PMC9445013

[B7] Braga F. Morgado S. Roque F. Morgado M. (2021). The role of the hospital pharmacist in immunocellular therapy with chimeric antigen receptor (CAR) T cells. Drugs Ther. Perspect. 37 (9), 433–438. 10.1007/s40267-021-00857-8

[B8] Dannenfelser R. Allen G. M. VanderSluis B. Koegel A. K. Levinson S. Stark S. R. (2020). Discriminatory power of combinatorial antigen recognition in cancer T cell therapies. Cell. Syst. 11 (3), 215–228. 10.1016/j.cels.2020.08.002 32916097 PMC7814417

[B9] Egri N. Ortiz de Landazuri I. San Bartolomé C. Ortega J. R. Español-Rego M. Juan M. (2020). CART manufacturing process and reasons for academy-pharma collaboration. Immunol. Lett. 217, 39–48. 10.1016/j.imlet.2019.10.014 31669547

[B10] EMA/188757/2022 Kymriah (2022). EMA/188757/2022 Kymriah (tisagenlecleucel): an overview of Kymriah and why it is authorised in the EU. Available at: https://www.ema.europa.eu/en/documents/overview/kymriah-epar-medicine-overview_en.pdf.

[B11] Enejo Ben (2019). Changing gears to deliver CAR-T in your hospital: enabling operational readiness for CAR-T therapy delivery in a hospital. Available at: https://www.adlittle.com/en/insights/report/changing-gears-deliver-car-t-your-hospital.

[B12] European Commision (2021b). Accelerating development and improving access to CAR and TCR-engineered T cell therapy. Available at: https://cordis.europa.eu/project/id/945393/de.

[B13] European Commision (2021a). Artificial intelligence-driven, decentralized production for advanced therapies in the hospital. Available at: https://cordis.europa.eu/project/id/101016909/de.

[B14] European Commision (2019). Immune safety avatar: nonclinical mimicking of the immune system effects of immunomodulatory therapies. Available at: https://cordis.europa.eu/project/id/853988/de.

[B15] Fleuren L. M. Klausch T. L. T. Zwager C. L. Schoonmade L. J. Guo T. Roggeveen L. F. (2020). Machine learning for the prediction of sepsis: A systematic review and meta-analysis of diagnostic test accuracy. Intensive Care Med. 46 (3), 383–400. 10.1007/s00134-019-05872-y 31965266 PMC7067741

[B16] Giannini H. M. Ginestra J. C. Chivers C. Draugelis M. Hanish A. Schweickert W. D. (2019). A machine learning algorithm to predict severe sepsis and septic shock: development, implementation, and impact on clinical practice. Crit. Care Med. 47 (11), 1485–1492. 10.1097/CCM.0000000000003891 31389839 PMC8635476

[B17] Geethakumari P. R. Ramasamy D. P. Dholaria B. Berdeja J. Kansagra A. (2021). Balancing quality, cost, and access during delivery of newer cellular and immunotherapy treatments. Curr. Hematol. Malig. Rep. 16 (4), 345–356. 10.1007/s11899-021-00635-3 34089485 PMC8179081

[B18] Ghassemi S. Martinez-Becerra F. J. Master A. M. Richman S. A. Heo D. Leferovich J. (2020). Enhancing chimeric antigen receptor T cell anti-tumor function through advanced media design. Mol. Ther. Methods Clin. Dev. 18, 595–606. 10.1016/j.omtm.2020.07.008 32775494 PMC7397397

[B19] Gil L. Grajek M. (2022). Artificial intelligence and chimeric antigen receptor T-cell therapy. Acta Haematol. Pol. 53 (3), 176–179. 10.5603/ahp.a2022.0019

[B20] Haag C. (2022). “Treatment coverage and reimbursement,” in The EBMT/EHA CAR-T cell handbook. Editors Kröger N. Gribben J. Chabannon C. Yakoub-Agha I. Einsele H. (Cham: CH), 229–230. 36122063

[B21] Haan K. (2023). 24 top AI statistics and trends in 2023: forbes advisor. Available at: https://www.forbes.com/advisor/business/ai-statistics/.

[B22] Harris E. Elmer J. J. (2021). Optimization of electroporation and other non-viral gene delivery strategies for T cells. Biotechnol. Prog. 37 (1), e3066. 10.1002/btpr.3066 32808434

[B23] Hort S. Herbst L. Bäckel N. Erkens F. Niessing B. Frye M. (2022). Toward rapid, widely available autologous CAR-T cell therapy - artificial intelligence and automation enabling the smart manufacturing hospital. Front. Med. 9, 913287. 10.3389/fmed.2022.913287 PMC9207622 35733863

[B24] Iyer R. K. Bowles P. A. Kim H. Dulgar-Tulloch A. (2018). Industrializing autologous adoptive immunotherapies: manufacturing advances and challenges. Front. Med. 5, 150. 10.3389/fmed.2018.00150 PMC5974219 29876351

[B25] Kamal-Bahl S. Puckett J. T. Bagchi I. Miller-Sonet E. Huntington S. F. (2022). Barriers and solutions to improve access for chimeric antigen receptor therapies. Immunotherapy. 10.2217/imt-2022-0037 35621253

[B26] Kühl N. Goutier M. Hirt R. Satzger G. (2020). Machine learning in artificial intelligence: towards a common understanding 2020. Available at: https://arxiv.org/pdf/2004.04686.

[B27] Kühl N. Schemmer M. Goutier M. Satzger G. (2022). Artificial intelligence and machine learning. Electron Mark. 32 (4), 2235–2244. 10.1007/s12525-022-00598-0

[B28] Kühn A. Joppen R. Reinhart F. Röltgen D. Enzberg S. von Dumitrescu R. (2018). Analytics canvas ‐ A framework for the design and specification of data Analytics projects. Procedia CIRP 70, 162–167. 10.1016/j.procir.2018.02.031

[B29] Kymriah (2018). 26/04/2023 Kymriah - emea/H/C/004090 - r/0068. Available at: https://www.ema.europa.eu/en/documents/product-information/kymriah-epar-product-information_en.pdf.

[B30] Le S. Hoffman J. Barton C. Fitzgerald J. C. Allen A. Pellegrini E. (2019). Pediatric severe sepsis prediction using machine learning. Front. Pediatr. 7, 413. 10.3389/fped.2019.00413 31681711 PMC6798083

[B31] Lee M. Lee Y-H. Song J. Kim G. Jo Y. Min H. (2020). Deep-learning-based three-dimensional label-free tracking and analysis of immunological synapses of CAR-T cells. Elife 9, e49023. 10.7554/eLife.49023 33331817 PMC7817186

[B32] Liberini V. Laudicella R. Capozza M. Huellner M. W. Burger I. A. Baldari S. (2021). The future of cancer diagnosis, treatment and surveillance: A systemic review on immunotherapy and immuno- pet radiotracers. Molecules 26 (26), 2201. 10.3390/molecules26082201 33920423 PMC8069316

[B33] Marofi F. Motavalli R. Safonov V. A. Thangavelu L. Yumashev A. V. Alexander M. (2021). CAR T cells in solid tumors: challenges and opportunities. Stem Cell. Res. Ther. 12 (1), 81. 10.1186/s13287-020-02128-1 33494834 PMC7831265

[B34] Maslej N. Fattorini L. Brynjolfsson E. Etchemendy J. Ligett K. Lyons T. (2023). The AI index 2023 annual report. Stanford, CA, April: Institute for Human-Centered AI, Stanford University.

[B35] Mösch A. Raffegerst S. Weis M. Schendel D. J. Frishman D. (2019). Machine learning for cancer immunotherapies based on epitope recognition by T cell receptors. Front. Genet. 10, 1141. 10.3389/fgene.2019.01141 31798635 PMC6878726

[B36] Moutsatsou P. Ochs J. Schmitt R. H. Hewitt C. J. Hanga M. P. (2019). Automation in cell and gene therapy manufacturing: from past to future. Biotechnol. Lett. 41 (11), 1245–1253. 10.1007/s10529-019-02732-z 31541330 PMC6811377

[B37] Naghizadeh A. Tsao W-C. Hyun Cho J. Xu H. Mohamed M. Li D. (2022). *In vitro* machine learning-based CAR T immunological synapse quality measurements correlate with patient clinical outcomes. PLoS Comput. Biol. 18 (3), e1009883. 10.1371/journal.pcbi.1009883 35303007 PMC8955962

[B38] Novartis (2023). Novartis. Übersicht behandelnder CAR-T zentren in deutschland 2023. Available at: https://www.novartis.com/de-de/uebersicht-behandelnder-car-t-zentren-deutschland.

[B39] O'Reilly M. A. Malhi A. Cheok K. P. L. Ings S. Balsa C. Keane H. (2023). A novel predictive algorithm to personalize autologous T-cell harvest for chimeric antigen receptor T-cell manufacture. Cytotherapy 25 (3), 323–329. 10.1016/j.jcyt.2022.10.012 36513573

[B40] Pan K. Farrukh H. Chittepu V. C. S. R. Xu H. Pan C-X. Zhu Z. (2022). CAR race to cancer immunotherapy: from CAR T, CAR NK to CAR macrophage therapy. J. Exp. Clin. Cancer Res. 41 (1), 119. 10.1186/s13046-022-02327-z 35361234 PMC8969382

[B41] Paul-Ehrlich-Institut (2023). Gene therapy medicinal products. Available at: https://www.pei.de/EN/medicinal-products/atmp/gene-therapy-medicinal-products/gene-therapy-node.html.

[B42] Rees J. H. (2022). “Management of immune effector cell-associated neurotoxicity syndrome,” in The EBMT/EHA CAR-T cell handbook. Editors Kröger N. Gribben J. Chabannon C. Yakoub-Agha I. Einsele H. (Cham: CH), 141–146. 36122055

[B43] Reyes S. J. Durocher Y. Pham P. L. Henry O. (2022). Modern sensor tools and techniques for monitoring, controlling, and improving cell culture processes. Processes 10 (2), 189. 10.3390/pr10020189

[B44] Sallam R. Steenstrup K. Eriksen L. Jacobson S. (2014). Industrial Analytics revolutionizes big data in the digital business. Available at: https://www.gartner.com/en/documents/2826118.

[B45] Solbach T. Kremer M. Stangier M. (2020). CAR-T-Zelltherapien in deutschland: Eine zwischenbilanz 2020.

[B46] Stock S. Schmitt M. Sellner L. (2019). Optimizing manufacturing protocols of chimeric antigen receptor T cells for improved anticancer immunotherapy. Int. J. Mol. Sci. 20 (24), 6223. 10.3390/ijms20246223 31835562 PMC6940894

[B47] Sugimoto K. (2019). Machine learning-driven label-free cell sorting for CAR-T cell manufacturing. Cytotherapy 21 (5), S39. 10.1016/j.jcyt.2019.03.376

[B48] Tang S. Chappell G.t T. Mazzoli A. Tewari M. Choi S. W. Wiens J. (2020). Predicting acute graft-versus-host disease using machine learning and longitudinal vital sign data from electronic health records. JCO Clin. Cancer Inf. 4 (4), 128–135. 10.1200/CCI.19.00105 PMC7049247 32083957

[B49] Tedesco V. E. Mohan C. (2021). Biomarkers for predicting cytokine release syndrome following CD19-targeted CAR T cell therapy. J. Immunol. 206 (7), 1561–1568. 10.4049/jimmunol.2001249 33692146

[B50] Vormittag P. Gunn R. Ghorashian S. Veraitch F. S. (2018). A guide to manufacturing CAR T cell therapies. Curr. Opin. Biotechnol. 53, 164–181. 10.1016/j.copbio.2018.01.025 29462761

[B51] Wörmann B. (2020). Qualitätsgesicherte durchführung in deutschland: Stand 5/2020 2020.

[B52] Wu Y. Y. Yong D. Naing M. W. (2018). Automated cell expansion: trends and outlook of critical technologies. Cell. Gene Ther. Insights 4 (9), 843–863. 10.18609/cgti.2018.087

